# Ideal serum non-ceruloplasmin bound copper prediction for long-term treated patients with Wilson disease: a nomogram model

**DOI:** 10.3389/fmed.2023.1275242

**Published:** 2023-11-03

**Authors:** Zhuang Tao, Pingping Yang, Jiafeng Zhou, Rui Wang, Zhenzhen Jiang, Hui Han, Meixia Wang

**Affiliations:** ^1^Encephalopathy Center, The First Affiliated Hospital of Anhui University of Chinese Medicine, Hefei, Anhui, China; ^2^Graduate School, Anhui University of Chinese Medicine, Hefei, Anhui, China

**Keywords:** Wilson disease, serum NCBC, clinical predictive model, retrospective study, concordance index

## Abstract

**Purpose:**

This study aimed to explore the factors associated with the optimal serum non-ceruloplasmin bound copper (NCBC) level and develop a flexible predictive model to guide lifelong therapy in Wilson disease (WD) and delay disease progression.

**Methods:**

We retrospectively collected clinical data from 144 patients hospitalized in the Encephalopathy Center of the first affiliated hospital of Anhui University of Chinese Medicine between May 2012 and April 2023. Independent variables were selected using variate COX and LASSO regressions, followed by multivariate COX regression analysis. A predictive nomogram was constructed and validated using the concordance index (C-index), calibration curves, and clinical decision curve analysis, of which nomogram pictures were utilized for model visualization.

**Results:**

A total of 61 (42.36%) patients were included, with an average treatment duration of 55.0 (range, 28.0, 97.0) months. Multivariate regression analysis identified several independent risk factors for serum NCBC level, including age of diagnosis, clinical classification, laminin liver stiffness measurement, and copper to zinc ratio in 24-h urinary excretion. The C-index indicated moderate discriminative ability (48 months: 0.829, 60 months: 0.811, and 72 months: 0.819). The calibration curves showed good consistency and calibration; clinical decision curve analysis demonstrated clinically beneficial threshold probabilities at different time intervals.

**Conclusion:**

The predictive nomogram model can predict serum NCBC level; consequently, we recommend its use in clinical practice to delay disease progression and improve the clinical prognosis of WD.

## Introduction

Wilson disease (WD) is an autosomal recessive inherited disorder of copper metabolism disorder caused by mutations in *ATP7B*, which encodes a copper-transporting P-type ATPase (ATP7B). Excessive copper deposition in various organs leads to a range of manifestations, such as hepatic injury, neurological symptomatology, and the presence of corneal Kayser-Fleischer rings (K-F rings) (1). Clinical treatment of WD typically involves the use of anticopper agents, such as D-Penicillamine (DPA), trientine, tetrathiomolybdate (TM), zinc, and sodium dimercaptosuccinate (DMPS). Approximately 2–6 months following the initiation of therapy, maintenance treatment of chelators and/or zinc therapy could be used for patients with WD. Further, lifelong therapy for patients with WD varies depending on clinical phenotype and efficacy, as result of ~10–50% risk of worsened neurologic symptoms or early and later sensitivity reactions during the initial and life-longed phase of treatment, the use of Dimercaptosuccinic acid (DMSA) and zinc is recommended for WD in China (2, 3). Lifelong therapy is closely related to the clinical progression and prognosis for WD. Poor medication compliance increases the risk of symptom recurrence and can even lead to liver failure, necessitating liver transplantation for survival (4).

Copper is an essential trace element in the human body, serving as a cofactor or critical component of over 30 enzymes and copper-containing proteins (5). It plays a vital role in physiological processes of mitochondrial metabolism and the maintenance of redox balance (6, 7). In healthy individuals, serum copper exists in two forms: ceruloplasmin-bound copper and non-ceruloplasmin bound copper (NCBC). Ceruloplasmin-bound copper constitutes ~85–95% (0.85–0.95 mg/L) of total serum copper and is covalently bound to ceruloplasmin. Copper in form of NCBC accounts for ~5–15% (0.05–0.15 mg/L) of total serum copper and loosely binds to albumin and other molecules in the body (8).

NCBC has been proposed as an indicator for monitoring therapy in WD. In untreated patients, serum NCBC levels are significantly elevated (up to 0.25 mg/L) compared to the normal range of ~0.1–0.15 mg/L in healthy individuals; however, regular anti-copper treatment could normalize these levels. A serum NCBC level exceeding 0.15 mg/L indicates poor compliance and may require additional interventions for WD management. Conversely, levels below 0.05 mg/L might indicate systemic copper depletion, suggesting overtreatment in some patients (4). Based on our present knowledge, there is a notable correlation between serum NCBC levels and the progression of neurological symptoms during the initial treatment of WD. Copper toxicity and abnormal deposition of serum NCBC both contribute to the disease progression in WD (9, 10).

In a previous study, Chenling et al. developed a predictive nomogram for early diagnosis and prevention of liver fibrosis in patients with WD who have abnormal lipid metabolism (11). Zhou Zheng et al. revealed predictive variables for portal venous system thrombosis after splenectomy by constructing an effective visual risk prediction model (12). However, to our knowledge, clinical predictive models have not been applied to predict the achievement of the ideal level of serum NCBC in patients with WD. Therefore, the current study explores the risk factors associated with suboptimal levels of serum NCBC and constructs a LASSO-nomogram model as this model facilitates patient monitoring and provides more accurate information about treatment compliance, enabling the flexible adjustment of lifelong therapy for Wilson disease (WD) and maximizing the delay of disease progression.

## Materials and methods

### Study design

We collected clinical data from patients hospitalized in the Encephalopathy Center of the first affiliated hospital of Anhui University of Chinese Medicine from January 2023 to April 2023. This study was approved by the Ethics Committee of the First Affiliated Hospital of Anhui University of Chinese Medicine and conducted in accordance with the principles outlined in the Declaration of Helsinki. Our analysis and reporting of the study followed the guidelines outlined in the Transparent Reporting of a multivariate prediction model for Individual Prognosis Or Diagnosis (TRIPOD): Explanation and Elaboration (13).

### Participants' enrollment

**Figure 1:**
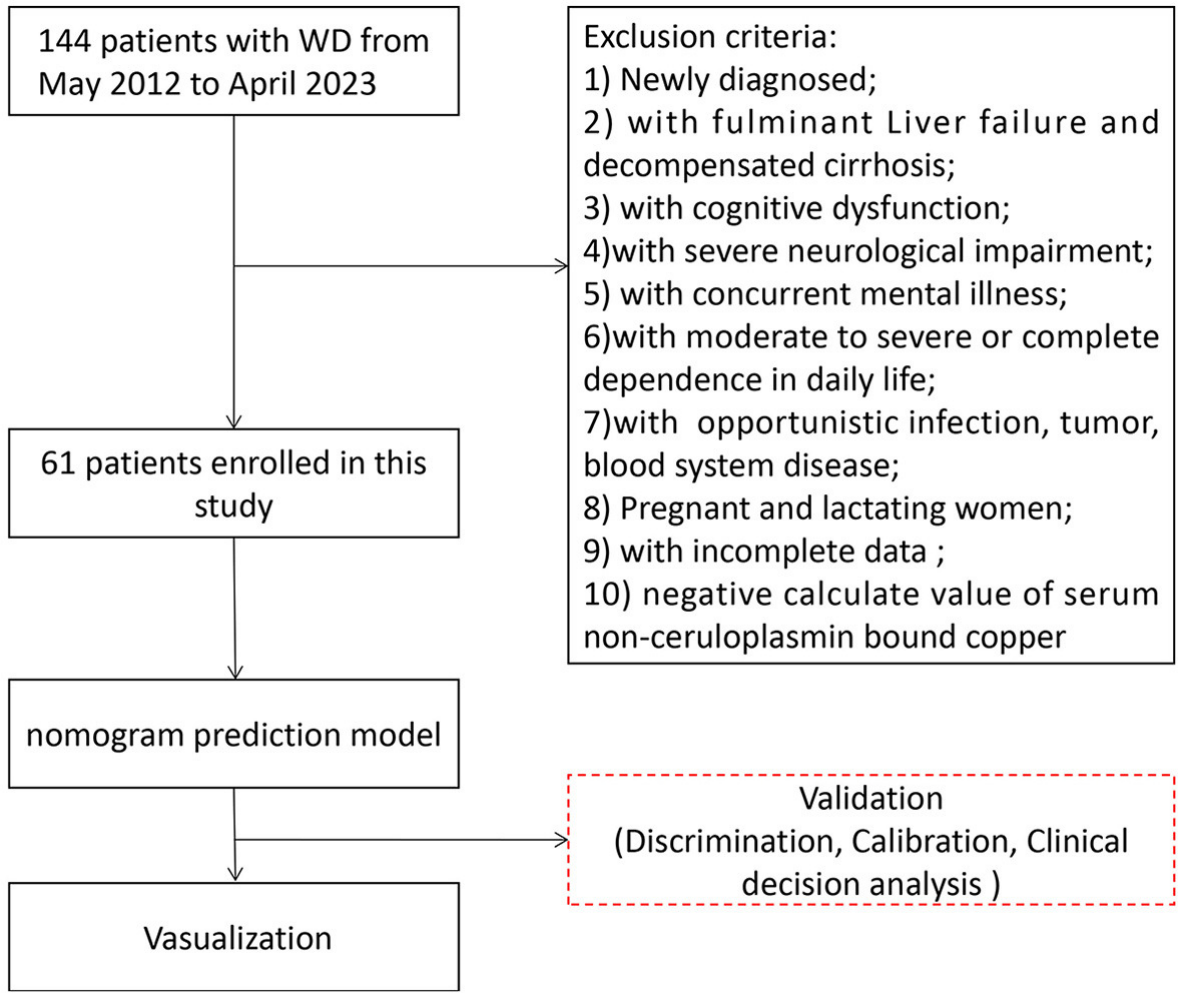
Participants flowchart in our study. A total of 144 patients were enrolled in our study between May 2012 and April 2023; 61 patients with WD were included in the statistical analysis.

We retrospectively collected clinical data from 144 patients hospitalized in the Encephalopathy Center of the first affiliated hospital of Anhui University of Chinese Medicine between May 2012 and April 2023. Sixty-one patients with WD were included in this study. All enrolled patients were diagnosed according to the guidelines outlined in the “EASL Clinical Practice Guidelines: Wilson's disease from the European Association for the Study of the Liver” and the “Diagnosis and Treatment of Wilson Disease: An Update from the American Association for the Study of Liver Disease.” The following exclusion criteria were applied: (1) Newly diagnosed patients; (2) Fulminant liver failure (with or without hemolytic anemia) and decompensated cirrhosis; (3) Cognitive dysfunction (Mini-Mental State Scale score of ≤ 22 or Montreal Cognitive Assessment Scale score of <26); (4) Severe neurological impairment, such as torsion spasms; (5) Concurrent mental illness; (6) Moderate to severe or complete dependence in daily life (Barthel index rating scale ≤ 70 points); (7) Serious diseases (opportunistic infections, tumors, blood system diseases); (8) Pregnancy and lactation; (9) Incomplete data required for the study; 10) Negative serum NCBC values. Please refer to Figure 1 for the participant flowchart.

### Data collection

Two investigators independently extracted the following data: (1) Basic information: age, sex, marital status, allergies, disease course, clinical classification, history of splenectomy, treatment duration, and treatment compliance; (2) Biochemical indicators: white blood cell (WBC), red blood cell (RBC), hemoglobin (HGB), platelet (PLT), reticulocyte (Ret), alanine aminotransferase (ALT), aspartate aminotransferase (AST), γ-glutamyl transpeptidase (GGT), blood urea nitrogen (BUN), serum creatinine (Scr), cystatin C, type IV collagen (CIV), hyaluronic acid (HA), laminin (LN), type III procollagen peptide (PIIINP), and liver stiffness measurement (LSM); (3) Copper biochemical indicators: serum NCBC, baseline serum NCBC, serum copper, ceruloplasmin, 24-h urinary copper excretion, copper to zinc ratio in 24-h urinary excretion, and copper to zinc ratio in serum; (4) Microelements indicators: serum zinc, serum calcium, serum magnesium, 24-h urinary zinc excretion, 24-h urinary calcium excretion, and 24-h urinary magnesium excretion.

### Statistical analysis

We performed statistical analysis using R software (version 4.1.1; http://www.r-project.org). The ideal level of serum NCBC was defined as 0.05–0.15 mg/L (14). Univariate COX and LASSO regression analyses were conducted to identify potential clinical variables. Variables with *P* < 0.1 were further analyzed using the multivariate COX regression analysis. For the construction of the nomogram models, the dependent variable was non-ceruloplasmin-bound copper level, and the independent variables included basic information, biochemical indicators, copper biochemical indicators, and microelements indicators. The concordance index (C-index), calibration curves, and clinical decision curve analysis (DCA) were applied to assess the validity of the nomogram model. Nomogram pictures were created to visualize the model (15, 16).

## Results

### Patient characteristics

Our study included 61 patients with WD, of which 30 patients (49.2%) were classified as the ideal level group based on their serum NCBC and 31 patients (50.8%) as the unideal group. The patients' mean age was 30.6 ± 9.15 years, including 35 males (57.4%) and 26 females (42.6%), with 10.00 (6.00, 15.00) months of disease course. The treatment duration was 55.00 (28.00, 97.00) months, including 43 patients (70.50%) in good compiance and 18 patients (29.50%) in bad compliance, of which 16 patients (26.20%) have undergone splenectomy. Among the patients, there were three clinical classifications: 27 patients (44.3%) presented with neurological symptomatology, 7 patients (11.50%) exhibited hepatic injury, and 27 patients (44.3%) were classified as mixed hepatic-neurologic type patients. The average treatment duration was 55.0 (range, 28.0, 97.0) months. Details of the included participants are shown in Table 1.

**Table 1:** Characteristics of the included patients.

**Characteristic**	**Included patients (*n* = 61)**
**Sex**	
Male	35 (57.40%)
Female	26 (42.60%)
**Age**	30.60 ± 9.15
**Marital status**	
Unmarried/divorce	33 (54.10%)
Married/cohabitation	28 (45.90%)
**Allergy**	
Yes	18 (29.50%)
No	43 (70.50%)
**Splenectomy**	
Yes	16 (26.20%)
No	45 (73.80%)
**Group**	
Acceptable	30 (49.20%)
Unacceptable	31 (50.80%)
**Compliance**	
Good compliance	43 (70.50%)
Bad compliance	18 (29.50%)
**Clinical classification**	
Hepatic injury	7 (11.50%)
Neurological symptomatology	27 (44.30%)
Mixed hepatic-neurologic type	27 (44.30%)
**Disease course (months)**	10.00 (6.00, 15.0)
**Treatment duration (months)**	55.0 (28.0, 97.0)

### Univariate LASSO regression analysis

**Figure 2:**
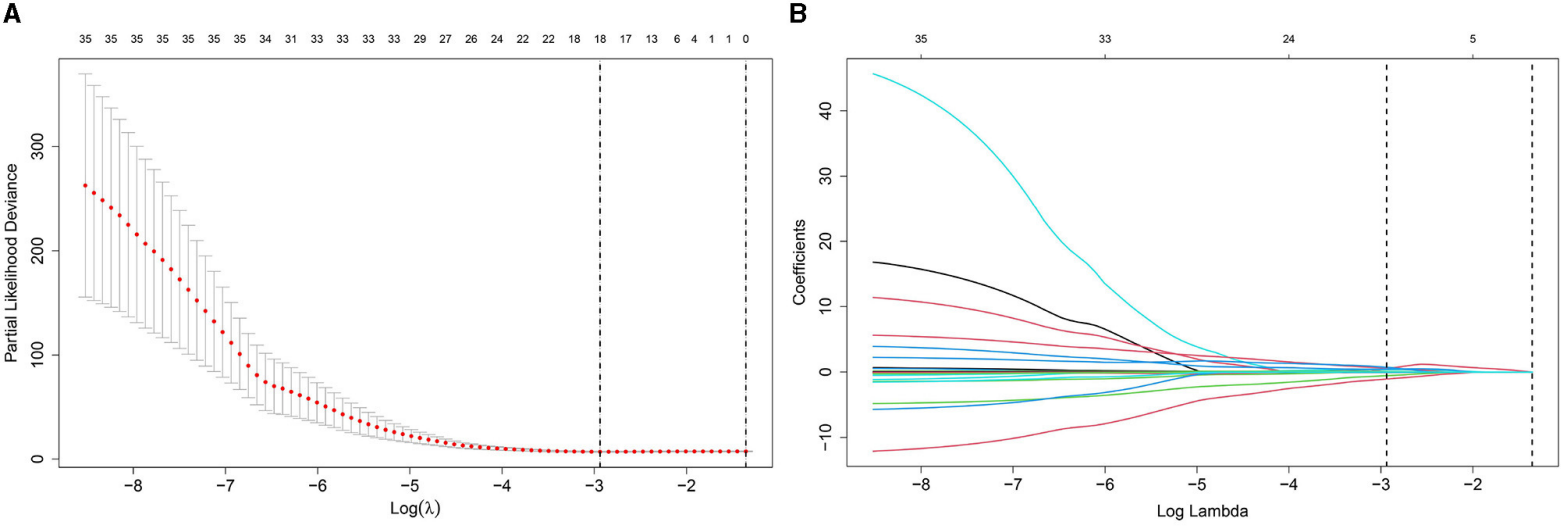
Diagram of univariate LASSO regression analysis. **(A)** Log_lambda and path coefficient diagram from LASSO regression analysis. **(B)** Diagram of CV_LASSO. Sex, age at diagnosis, marital status, clinical classification, disease course, compliance, WBC, RBC, ALT, LN, LSM, Scr, 24-h urinary zinc excretion, and copper to zinc ratio in 24-h urinary excretion were related factors for serum NCBC levels.

We selected clinical variables by performing univariate LASSO regression analysis. The analysis identified several related factors influencing serum NCBC levels, including sex, age, marital status, clinical classification, disease course, compliance, WBC, RBC, ALT, LN, LSM, Scr, 24-h urinary zinc excretion, and copper to zinc ratio in 24-h urinary excretion. Details regarding the LASSO regression analysis on these related factors are presented in Figure 2.

### Multivariate COX regression analysis

**Figure 3:**
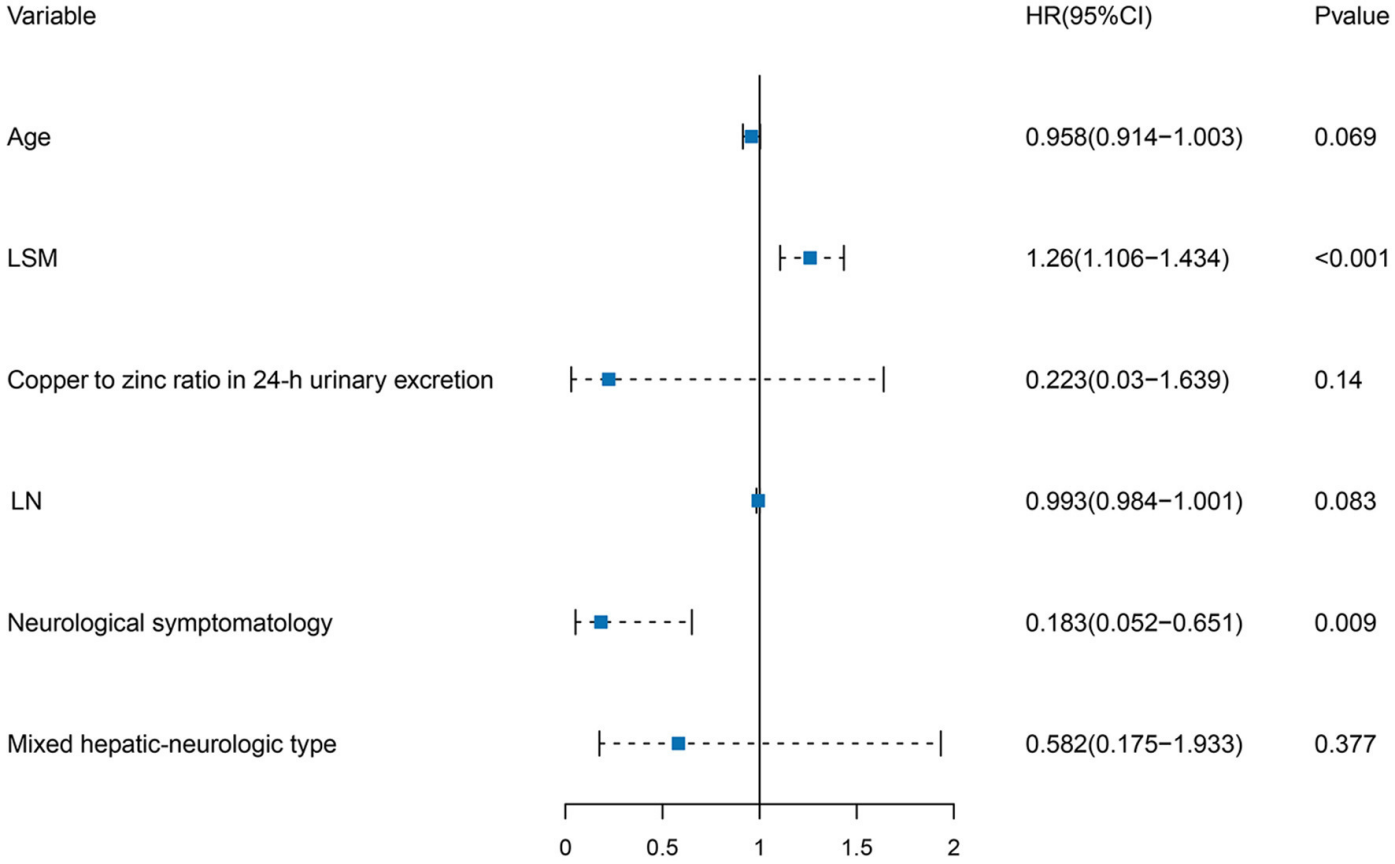
Forest plot of independent risk factors in multivariate COX regression analysis. Age of diagnosis, clinical classification, LN, LSM, and copper to zinc ratio in 24-h urinary excretion were independent risk factors for serum NCBC levels.

Multivariate COX regression analysis was performed using the clinical variables selected from the univariate LASSO analysis. Age of diagnosis [HR, 0.958 (95% CI: 0.914–1.003)], clinical classification [HR, 0.183 (95% CI: 0.052–0.651), and HR, 0.582 (95% CI: 0.175–1.933)], LN [HR, 0.993 (95% CI: 0.984–1.001)], LSM [HR, 1.260 (95% CI: 1.106–1.434)], and copper to zinc ratio in 24-h urinary excretion [HR, 0.223 (95% CI: 0.030–1.639)] were identified as independent risk factors for serum NCBC levels. Details of the multivariate COX regression analysis are shown in Table 2 and Figure 3.

**Table 2:** Multivariate COX regression analysis of independent risk factors.

**Characteristics**	**B**	**SE**	**HR**	**95% CI**	**Z**	***P***
Age	−0.043	0.024	0.958	0.914–1.003	−1.821	0.069
LN	−0.007	0.004	0.993	0.984–1.001	−1.736	0.083
LSM	0.231	0.066	1.260	1.106–1.434	3.482	0.000
Copper to zinc ratio in 24-h urinary excretion	−1.499	1.017	0.223	0.030–1.639	−1.474	0.140
Clinical classification						
Neurological symptomatology	−1.696	0.647	0.183	0.052–0.651	−2.623	0.009
Mixed hepatic-neurologic type	−0.541	0.612	0.582	0.175–1.933	−0.883	0.377

### The nomogram prediction model

We constructed the nomogram model using the independent risk factors selected through multivariate logistic regression analysis. To validate the nomogram model, we used the C-index, calibration curves, and DCA.

### Concordance index (c-index)

**Figure 4:**
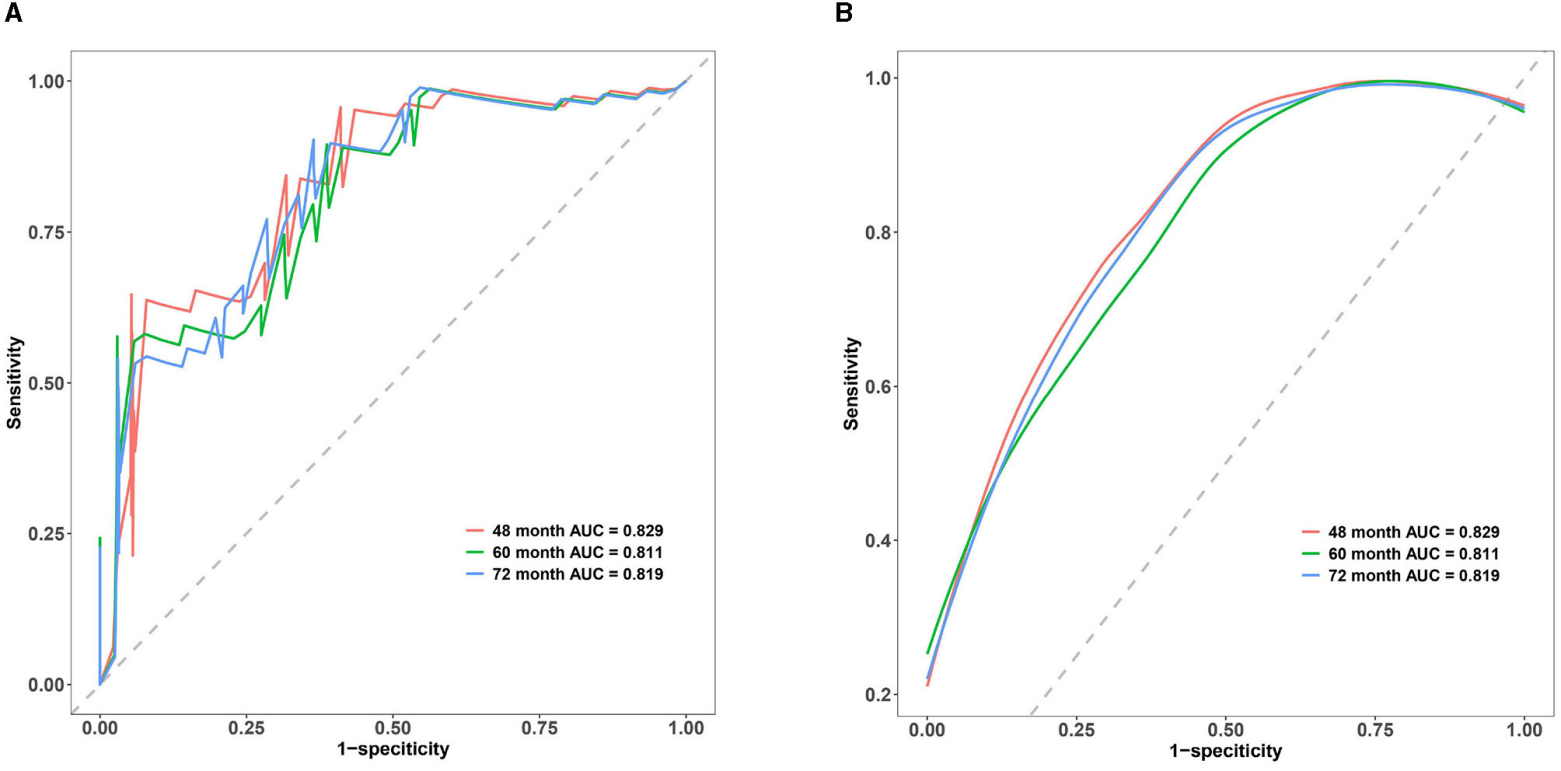
Multi-time ROC of the nomogram model. **(A)** Multi-time classic ROC of the nomogram model. **(B)** Multi-time smooth ROC of the nomogram model. The x-axis represents 1-specificity, whereas the y-axis represents sensitivity. The red line corresponds to the ROC curve for the 48-month treatment duration, the green line represents the 60-month treatment duration, and the blue line corresponds to the ROC curve for the 72-month treatment duration. The area below each line represents the respective area under the ROC curve for the nomogram model at 48-, 60-, and 72-month treatment durations. The C-index of the nomogram model in our study were demonstrated to be in an intermediate level of discrimination.

The C-index is commonly employed to assess the discrimination capability of the nomogram model. A C-index between 0.5 and 0.7 indicates a lower level of discrimination, whereas a C-index > 0.90 indicates a higher level of discrimination. When the C-index falls between 0.71 and 0.90, the discrimination level is considered intermediate. In our study, the C-index of the nomogram model demonstrated an intermediate level of discrimination (48 months: 0.829, 60 months: 0.811, and 72 months: 0.819). Details of the C-index are presented in Figure 4.

### Calibration curves

**Figure 5:**
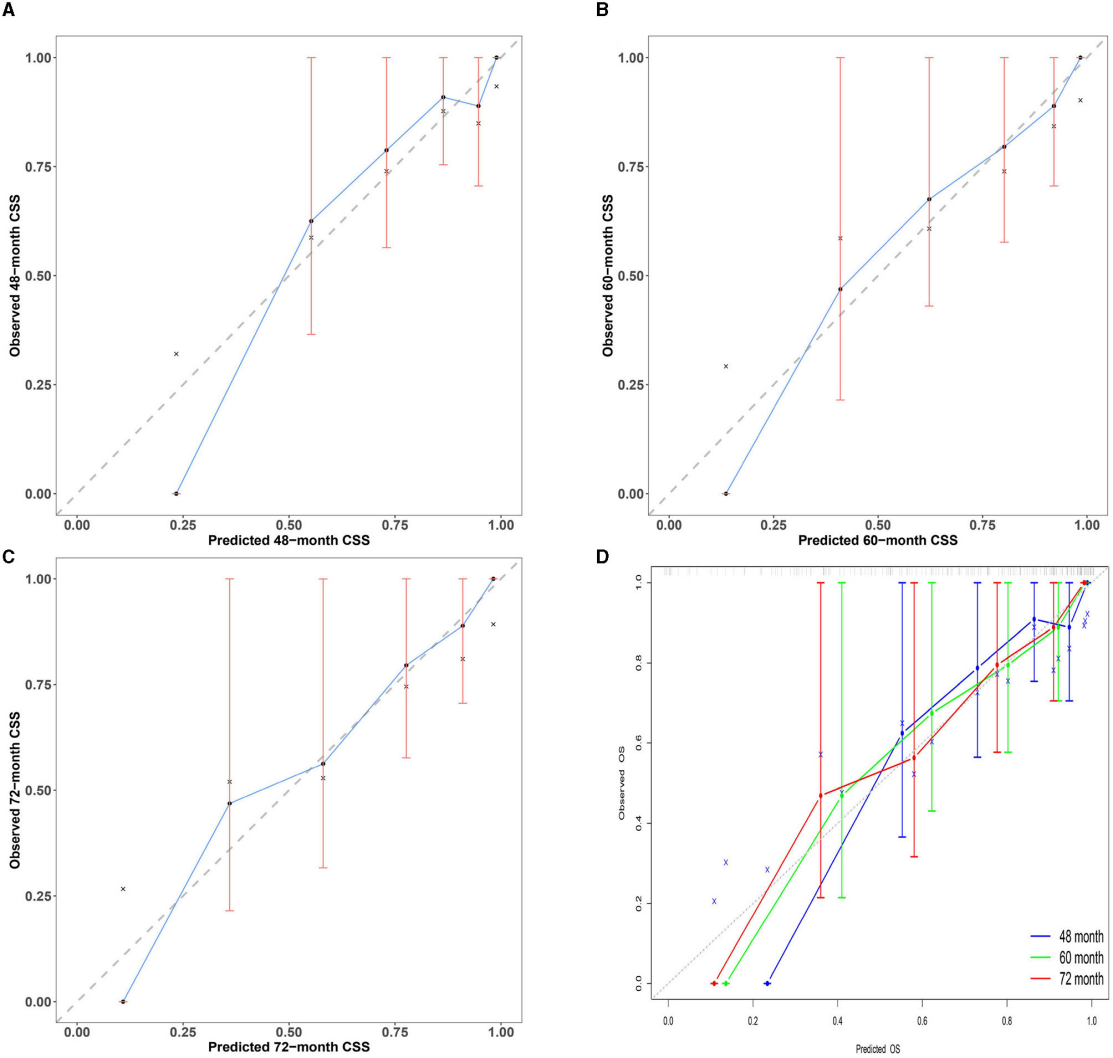
Multi-time calibration curves of the nomogram model. The x-axis represents the probability of the ideal level, and the y-axis represents the actual level of serum NCBC as predicted by the nomogram model. The black dashed line represents perfect prediction based on actual probability. The blue line represents the prediction of the nomogram model. The closer the interval is to the black dashed line, the better the performance of the nomogram model. **(A–C)** Calibration curves of the nomogram model for 48-, 60-, and 72-month treatment durations. **(D)** Overview calibration curves of multi-time the nomogram model. Calibration curves in our study were closely aligned with the reference line; this model has good consistency and calibration.

Calibration curves are essential for evaluating the accuracy of the nomogram model. This study's calibration curves closely align with the reference line, indicating that this model has good consistency and calibration. Details of calibration are presented in Figure 5.

### DCA

**Figure 6:**
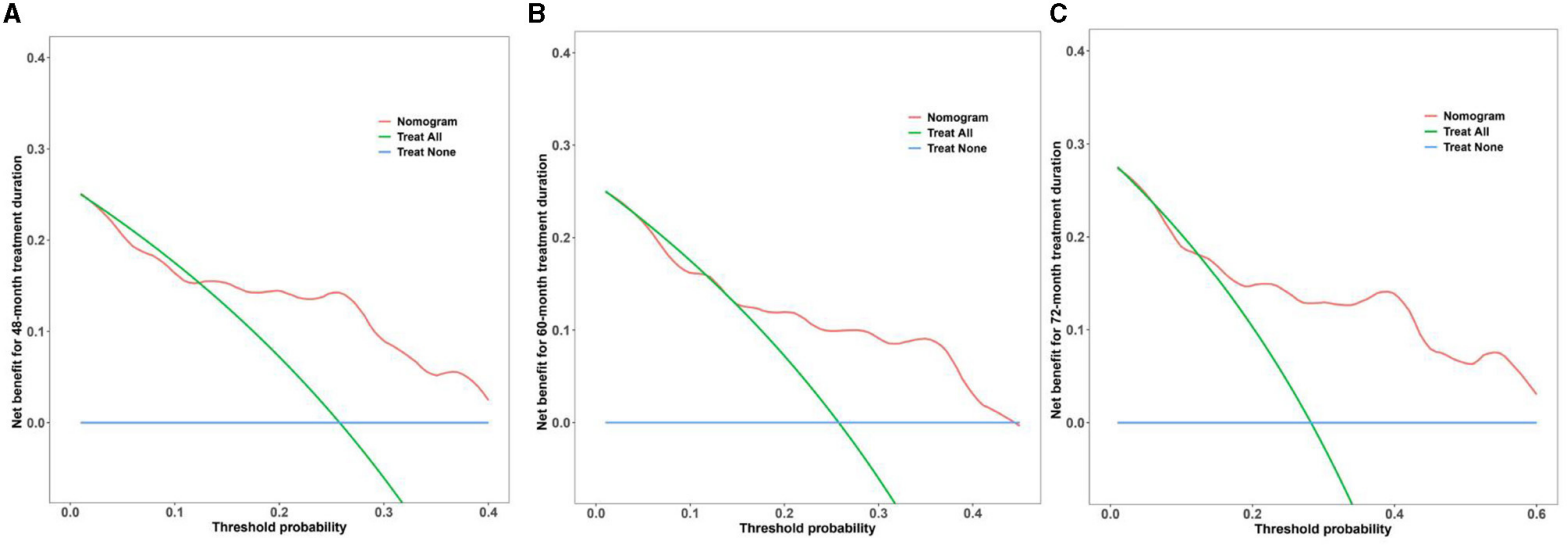
DCA curves of the nomogram model for multi-time treatment duration. **(A–C)** DCA curves of the nomogram model for 48-, 60-, and 72-month treatment durations. The x-axis represents the threshold probability, and the y-axis represents the net benefit of the nomogram model. The blue line represents the net benefit when all participants fail to achieve the ideal level of serum NCBC, whereas the green line represents the net benefit when all participants achieve the ideal level. The red line represents the net benefit according to the model's predictions. The model falls within a clinically beneficial range of threshold probabilities (48 months: 0.15–0.40, 60 months: 0.10–0.45, and 72 months: 0.15–0.60).

We evaluated the clinical usability and effectiveness of the nomogram model using DCA, which reflects the patient's net benefit. Our study demonstrates that the model falls within a clinically beneficial range of threshold probabilities (48 months: 0.15–0.40, 60 months: 0.10–0.45, and 72 months: 0.15–0.60). Details of the calibration are shown in Figure 6.

### Visualization of the nomogram model

**Figure 7:**
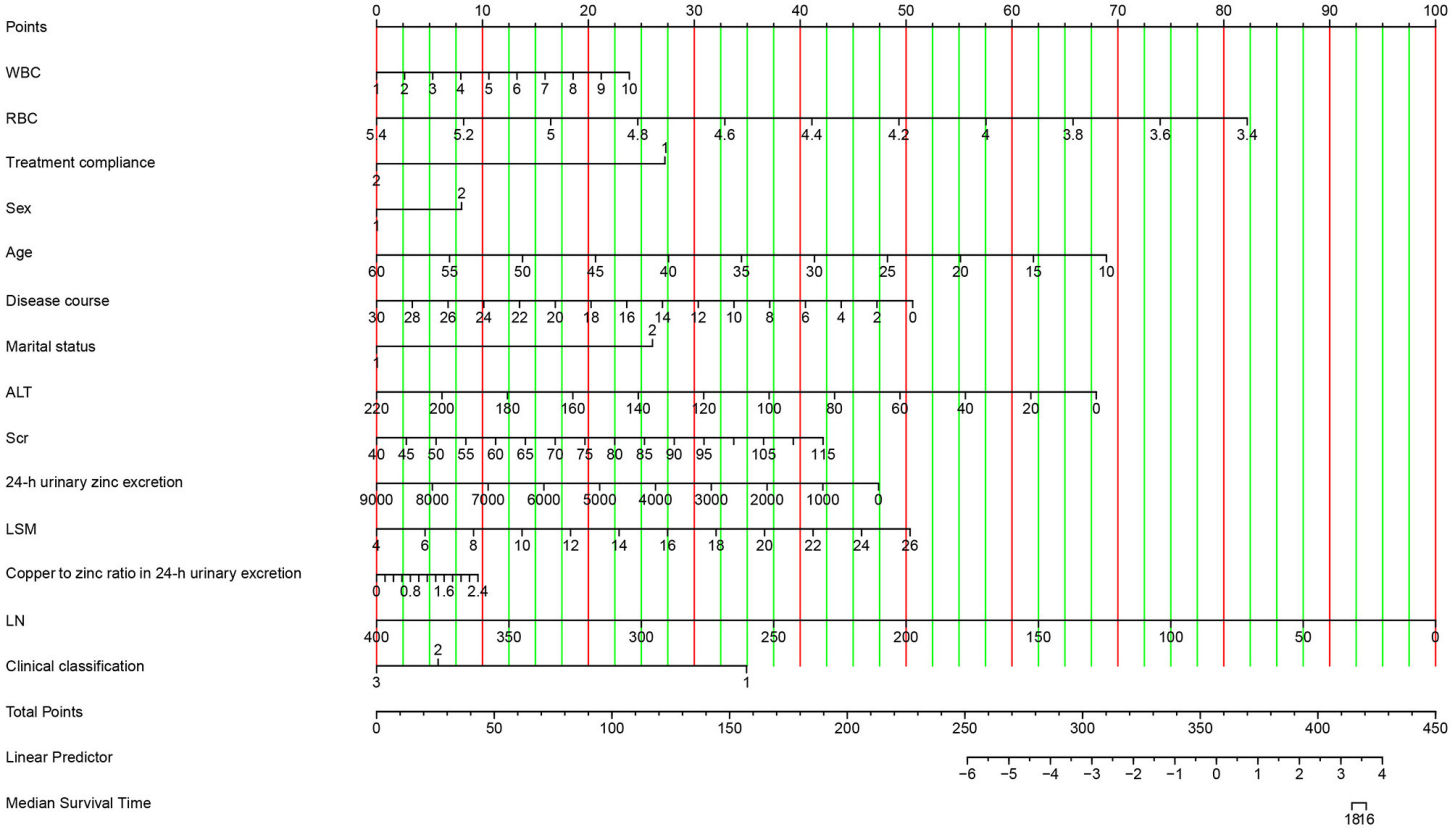
Visualization of the nomogram model. Variables are displayed on the left side, whereas scores are shown on the right side. The total score is calculated by adding the values of each variable. The occurrence probability of acceptable serum NCBC can be predicted using the linear predictor line.

The visualization of the nomogram model depicts variables on the left side of Figure 7. Using a point line, scores for multiple variables can be calculated based on their levels to obtain the total score, shown on the right side of Figure 7. We could draw a vertical line extending from the total points line down to the scale interval of the linear predictor line; the corresponding scale value represents the occurrence probability of achieving the ideal serum NCBC level. Details of the nomogram model visualization are shown in Figure 7.

### Reasonableness analysis

**Figure 8:**
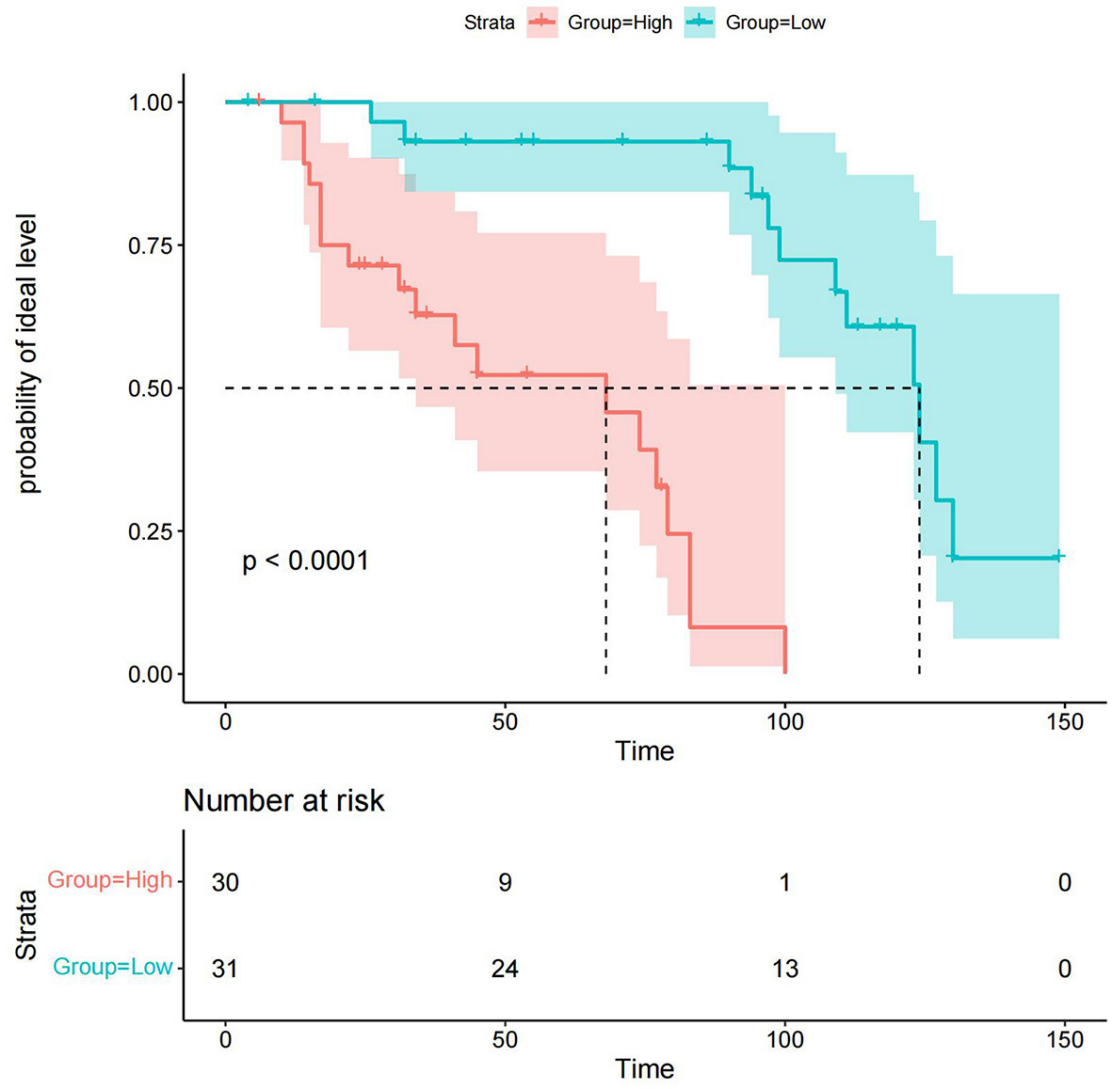
Reasonableness analysis of the nomogram model. Participants were divided into high or low-risk groups. The green line and area represent the probability of the ideal level and its 95% CI in the low-risk group, whereas the red line and area represent the probability of the ideal level and its 95% CI in the high-risk group. Participants in the low-risk group are more likely to achieve the ideal level of serum NCBC.

We performed a reasonableness analysis to evaluate the nomogram model, comparing occurrence probability of ideal level of serum NCBC by dividing participants into high or low-risk groups. Significant differences were observed in probability of an ideal NCBC level between two groups, demonstrating that participants in the low-risk group are more likely to obtain an ideal level of serum NCBC. Details of the reasonableness analysis of the nomogram model are presented in Figure 8.

## Discussion

In this study, we developed a predictive model to estimate the probability of achieving the ideal serum NCBC level in patients with WD. Age at diagnosis, clinical classification, LN, LSM, and copper to zinc ratio in 24-h urinary excretion were identified as the top five critical predictors in the nomogram model. The model demonstrated excellent discrimination and good calibration, clinical usability, and effectiveness.

The elevated level of serum NCBC resulting from copper metabolism disorders in the body is closely associated with the degree of copper toxicity (17). Research shows that patients with WD can exhibit serum NCBC levels as high as 0.5 mg/L due to reduced ceruloplasmin levels. A significant correlation exists between serum NCBC levels and the neurological deterioration in WD following copper removal treatment. Both the toxicity effects of serum NCBC and the abnormal deposition of copper contribute to the progression of the disease (18). Therefore, it is important for patients with WD to monitor their serum NCBC levels and ensure that they are maintained within an acceptable range. Magnetic resonance imaging of two patients with higher levels of NCBC developed neurological deterioration, shown in Supplementary material.

Age at diagnosis and clinical classification are predictive indicators for serum NCBC levels. Due to patients with WD experience pathological processes of hepatic copper accumulation, saturation, and release, the onset of disease is mostly between 5 and 40 years old (19, 20). Patients classified as neurological symptomatology or mixed hepatic-neurologic type tend to be older than hepatic injury type (21). Inconsistent with J.M. WALSHE's study (18), there was little difference in NCBC between patients with hepatic injury type and those with neurological symptomatology or mixed hepatic-neurologic type. In our study, neurological symptomatology or mixed hepatic-neurologic type patients would be more susceptible to unideal levels of NCBC, speculating that those patients have more decreased serum ceruloplasmin values and higher degree of human body damage.

The liver is one of the most commonly affected organs in patients with WD, with ~80% exhibiting varying degrees of liver damage (2, 22). In clinical practice, most symptoms of WD are non-specific symptoms of chronic liver disease, such as poor appetite, fatigue, drowsiness, and discomfort in the right rib cage. Approximately 10–30% of patients have chronic active hepatitis (23). A few patients may have asymptomatic hepatosplenomegaly during physical examination or only show increased liver transaminase levels (24). As the disease progresses, hepatic injury can gradually advance to liver fibrosis, eventually leading to cirrhosis. In clinical practice, complications such as gastrointestinal bleeding, hepatic encephalopathy, infection, portal vein thrombosis, or spongiform transformation often occur (25). Studies have shown that ~35–45% of patients, regardless of whether their main manifestation is liver damage, neuropsychiatric symptoms, or if they are asymptomatic, have cirrhosis at the time of WD diagnosis (26, 27). Consistent with previous research, lowering serum NCBC levels markedly decreased liver fibrosis indicators, inhibiting injury development and collagen deposition in hepatic (13), In our prediction model, LSM and LN were identified as related factors for achieving ideal serum NCBC levels, which serve as indicators of liver fibrosis that represents partial tissue repair response to chronic liver injury (28). It is wellestablished that liver fibrosis is a necessary pathological progression step bridging hepatic injury and cirrhosis (29, 30). By implementing effective interventions targeting liver fibrosis, it is possible to delay or even reverse the progression of hepatic injury to cirrhosis, thus improving the clinical prognosis for patients (31).

The 24-h urine copper level is an indirect reflection of serum NCBC levels and plays a crucial role in the diagnosis and treatment monitoring of WD (32, 33). For patients with WD undergoing copper excretion treatment, urinary copper serves as an important reference indicator for evaluating treatment efficacy, compliance, or adjusting drug dosage (18). In a retrospective study involving 158 subjects, it was observed that patients' 24-h urine copper level began to decrease after 4 weeks of treatment and showed a significant decrease at 8 weeks. After 1 year, the 24-h urine copper level of patients gradually stabilized at a lower level (168.77 ± 80.72 μg/24 h) following anti-copper treatment (34). During a 10-year clinical follow-up using zinc as a maintenance treatment regimen, it was found that the 24-h urinary copper levels decreased to 125 μg/24 h in most patients with WD within 6–12 months of therapy. The mean level decreased from 269 to 97 μg/24 h throughout the follow-up period (35). Additionally, in this study, the 24-h urine zinc level was shown to be an effective indicator for evaluating patient medication compliance. It is generally recommended that the average 24-h urine zinc level should not be below 2 mg/24 h and is typically around 3–4 mg/24 h (36). Limited studies have been conducted on the copper to zinc ratio in 24-h urinary excretion. However, compared to the 24-h urine copper alone, the copper to zinc ratio in 24-h urinary excretion shows a promising diagnostic parameter for WD. Analyzing both copper and zinc using the same instrument makes the copper to zinc ratio a more favorable option (33, 37). In this study, copper to zinc ratio in 24-h urinary excretion was identified as an independent influencing factor for serum NCBC levels in patients, reflecting both the patient's serum NCBC levels and medication compliance.

In our study, age at diagnosis and clinical classification are essential predictors in the nomogram model. Patients with neurological symptomatology or mixed hepatic-neurologic types were more likely to achieve an ideal serum NCBC level. We speculate that this observation is related to the age of diagnosis and the underlying disease pathogenesis in different clinical subtypes. The age of onset for WD varies among individuals and can range from 3 to 62 years, making age alone insufficient for excluding the disease (38). Renhua et al. reported that patients with predominantly hepatic WD manifestations had lower age of onset compared to those with neurological symptomatology and mixed hepatic-neurologic types (39). In a retrospective study involving 45 patients with WD who initially received anti-copper treatment, it was found that patients with predominantly hepatic disease had higher serum NCBC levels than those with neurological disease. The liver is the primary target organ for damage in WD as it has a relatively high free copper content compared with other organs or due to the higher sensitivity of participants with neurological disease to anti-copper drugs. Similarly, a study by Kumar et al. using a rat model exposed to CuSO_4_ found that oxidative stress induced by serum NCBC primarily affected the liver compared to the brain and kidney. This could be explained by the abnormal deposition of NCBC in the liver, leading to organ dysfunction (40).

The study has some limitations. Firstly, although we made efforts to collect comprehensive clinical data, some patients had their initial visit to our hospital more than 10 years ago, resulting in partial data incompleteness, which may have influenced the observation outcomes. Secondly, there are formula calculation method, graphite furnace atomic absorption spectrophotometry, and inductively coupled plasma mass spectrometry for NCBC evaluation at present, but there is no relatively mature method with better reliability and accuracy (10). Considering for practicality and operability in clinical practice, we calculated serum NCBC values relied on a formula. However, the presence of abnormal ceruloplasmin and serum copper levels exhibited by the disease can lead to negative serum-free copper values, which may introduce a risk of poor stability and large errors in the measurements. Then, there were a small number of subjects, and the validation was performed on the same group of subjects. limitation in our study, we prepare to try our best to apply our model to a subgroup of subjects randomly selected from future clinical. Furthermore, anti-copper drugs can bind to free copper in the body to form related compounds excreted in urine. This study did not account for the NCBC portion complexed by these related compounds. Finally, due to this study's retrospective nature and the lack of scoring data for clinical symptoms of patients with WD, we were unable to perform further analyses on the relationship between copper biochemical indicators and clinical symptom scoring that reflected treatment effectiveness and neurological deterioration, liver and kidney function, liver fibrosis indicators, neuroimaging indicators, and survival indicators.

In conclusion, age at diagnosis, clinical classification, LN, LSM, and copper to zinc ratio in 24-h urinary excretion emerged as independent predictors in the nomogram model for ideal serum NCBC in WD, which has notable practical value, with guiding timely treatment interventions, delaying disease progression and ultimately improving the clinical prognosis of patients with WD.

## Data availability statement

The original contributions presented in the study are included in the article/Supplementary material, further inquiries can be directed to the corresponding author.

## Ethics statement

The studies involving humans were approved by the Ethics Committee of The First Affiliated Hospital of Anhui University of Chinese Medicine. The studies were conducted in accordance with the local legislation and institutional requirements. Written informed consent for participation in this study was provided by the participants' legal guardians/next of kin.

## Author contributions

ZT: Conceptualization, Data curation, Methodology, Project administration, Writing—original draft. PY: Data curation, Resources, Writing—original draft. JZ: Resources, Software, Validation, Writing—original draft. RW: Resources, Software, Validation, Writing—original draft. ZJ: Methodology, Software, Visualization, Writing—original draft. HH: Formal analysis, Resources, Visualization, Writing—original draft. MW: Conceptualization, Formal analysis, Funding acquisition, Methodology, Project administration, Supervision, Writing—review & editing.
