# Supplementary material for: Ideal serum non-ceruloplasmin bound copper prediction for long-term treated patients with Wilson disease: a nomogram model
Source: Front Med (Lausanne). 2023 Nov 3;10:1275242. doi: 10.3389/fmed.2023.1275242 (PMC10656596; doi:10.3389/fmed.2023.1275242)
Supplement: Supplementary file 1 [file Data_Sheet_1.docx]

Supplementary Material

# 1 Supplementary Figures


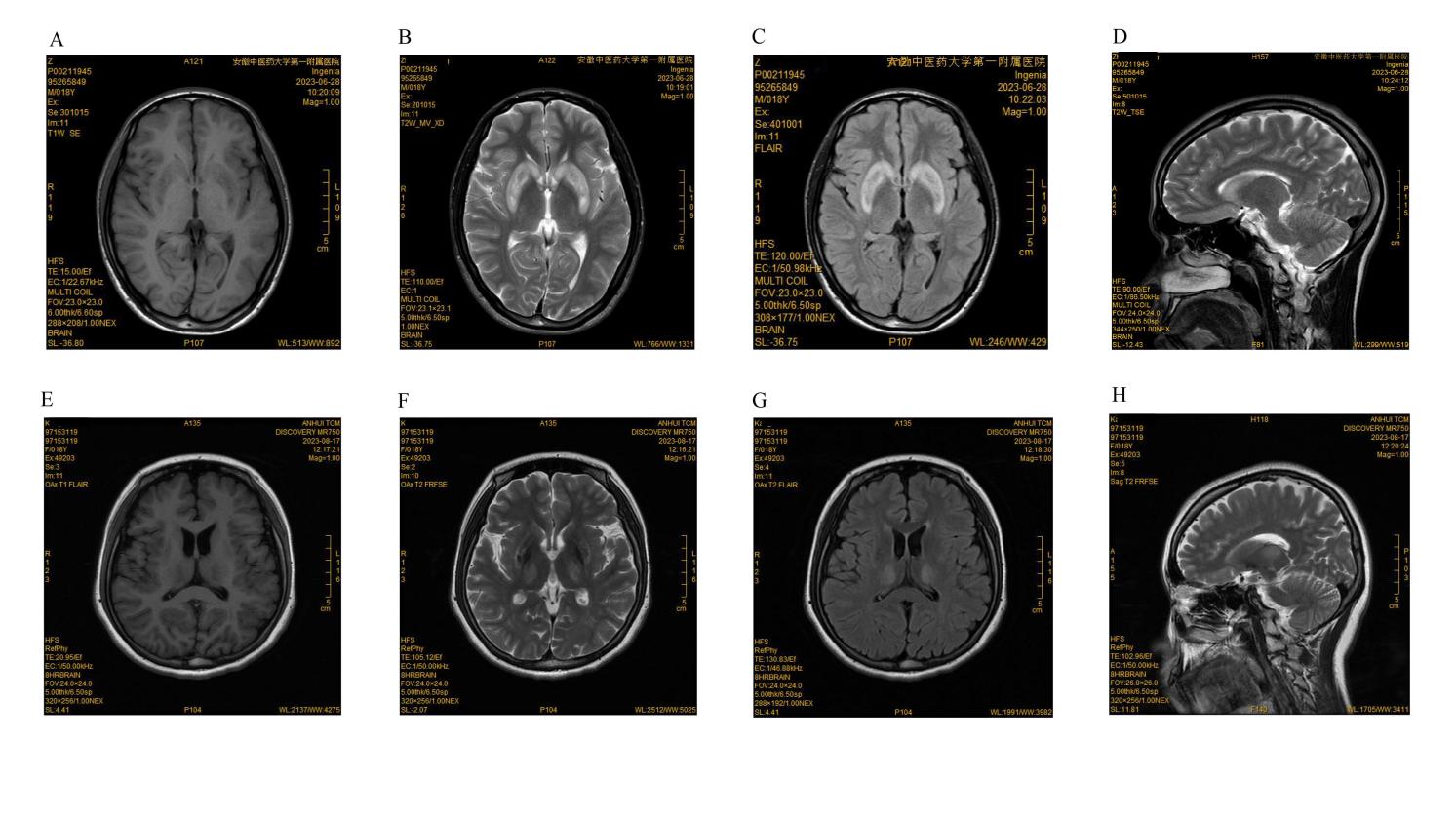


**Supplementary Figure 1.** Magnetic resonance imaging of two patients with higher NCBC. (A) - (D) ZHANG, male, 18 years old, with bilateral abnormal signal of lenticular nucleus, the serum non-ceruloplasmin bound copper (NCBC) level was 0.532 mg/L; (E) - (H) KANG, female, 18 years old, with bilateral abnormal signal of thalamus, the serum NCBC level was 0.541 mg/L. Both patients experienced neurological deterioration that characterized by trembling and dysarthria during treatment duration, it is inferred that patients with higher serum NCBC level should carefully choose or adjust anticopper agents.
